# Transcatheter arterial embolization in patients with neuroendocrine neoplasms related to liver metastasis with different blood supplies

**DOI:** 10.1002/cam4.6464

**Published:** 2023-08-17

**Authors:** Jianan Bai, Jinhua Song, Yang Zhang, Xiaolin Li, Lijun Yan, Ping Hu, Qiyun Tang

**Affiliations:** ^1^ Department of Geriatric Gastroenterology, The First Affiliated Hospital with Nanjing Medical University, Institute of Neuroendocrine Tumor Nanjing Medical University Nanjing China; ^2^ Department of Hepatobiliary Surgery The First Affiliated Hospital with Nanjing Medical University Nanjing China

**Keywords:** adverse events, blood supply, liver metastasis, neuroendocrine neoplasm, progression‐free survival, transhepatic artery embolization

## Abstract

**Purpose:**

Liver metastasis is one of the most important factors affecting the prognosis of patients with neuroendocrine neoplasms (NENs). Transhepatic artery embolization (TAE) is the main local treatment of NENs with liver metastasis (NENLM). This study aimed to elucidate the differences between pancreatic and rectal NENLM with a discrepancy in blood supply.

**Methods:**

A total of 32 patients with NENLM of different primary sites received 102 TAE treatments at our hospital. Clinical features, such as age, sex, World Health Organization (WHO) tumour grade and progression‐free survival (PFS), were compared between patients with pancreatic and rectal NENLM with different blood supplies. The total follow‐up time is 1–5 years.

**Results:**

There were 12 cases with tumours originating from the rectum or pancreas, respectively. Other tumour‐originated sites included the duodenum (two cases, 6.25%), the thymus and lung (four cases, 12.5%), and the unknown (two cases, 6.25%). The average age of patients was 51.59 years, and 17 (53.1%) were men. WHO grade 1, 2 or 3 tumours occurred in three (9.4%), 23 (71.9%) and six (18.7%) patients, respectively. Hepatic tumour burdens of low (<25%), middle (25%–50%) and high (>50%) levels were found in 13 (40.6%), eight (25%) and 11 (34.4%) patients, respectively. There were more patients with hypervascular pancreatic NENLM than with hypovascular rectal NENLM (*p* = 0.005). Tumour shrinkage in all cases with NENLM was 50% with an objective response rate of 37.5%, disease control rate of 75% and PFS of 12 months. Disease progression (*p* = 0.09), tumour shrinkage (*p* = 0.07) and death (*p* = 0.19) were more prominent in the pancreatic NENLM group than in the rectal NENLM group. Progression‐free survival was not reached in the pancreatic NENLM group, which was more prominent than in the rectal NENLM group (7 months; hazard ration, 0.22; 95% confidence interval, 0.07–0.76; *p* = 0.016). The main adverse events were abdominal pain (71.9%) and transaminase elevation (50%), which were more common in pancreatic NENLM than in rectal NENLM.

**Conclusions:**

Transhepatic artery embolization treatment is markedly effective and safe for treating NENLM, especially pancreatic NENLM.

## 1 INTRODUCTION

Neuroendocrine neoplasms (NENs) represent a group of highly heterogeneous tumours originating from neuroendocrine cells distributed throughout the body. According to the Surveillance, Epidemiology, and End Results database, the incidence of NENs has shown a significant upward trend in recent years, increasing 6.4 times to 6.98/100,000 from 1973 to 2012. The gastrointestinal tract and pancreas are the most common primary sites of NENs, accounting for nearly 60% of all cases.^1^, ^2^ Similarly, in China, pancreatic NENs (pNENs) and rectal NENs (rNENs) are the most common NENs, each accounting for 30% of all cases.^1^, ^2^, ^3^

According to the tumour proliferation index (Ki‐67) and mitotic data in WHO 2019, NENs are divided into the well‐differentiated neuroendocrine tumour (NET) grades G1, G2, G3 and poorly differentiated neuroendocrine carcinoma.^4^ The median overall survival (OS) of patients with NET is relatively longer than those with other malignant tumours, and it is even 25–30 years for patients with appendix and rectal NENs However, OS will be drastically shortened once distant metastasis occurs, even to less than 1 year. The liver is most prone to metastasis of NENs.^2^ Among all patients with metastasis, 65.2%–82% underwent liver metastasis, of which 65.5% were diffused to two or more lobes (type III) without indication for surgical resection.^5^, ^6^ The burden of liver tumour is a vital factor affecting the effect of drug and radionuclide therapies.^7^, ^8^ Therefore, controlling liver tumour burden has become the key strategy for treating liver metastatic NENs (NENLM).

Transcatheter arterial embolization (TAE) can block the blood supply of lesions and shrink tumours in hypervascularized primary and metastatic liver tumours, such as hepatocellular carcinoma.^9^, ^10^ Transhepatic artery embolization has been recommended by the National Comprehensive Cancer Network and European Neuroendocrine Tumor Society guidelines as an important local treatment of hypervascular NENLM.^11^, ^12^ However, there are few reports on the effects and adverse events of TAE in treating NENLM. Differences exist in the biological behaviours and clinical manifestations of patients with pancreatic NENLM and rectal NENLM, especially in terms of blood supply.^13^, ^14^ Transhepatic artery embolization might be used for hypovascular rectal NENLM because the guidelines recommended no other effective local treatments for liver metastases. However, few studies have reported the effect of TAE on hypovascular NENLM and the difference of TAE in treating pancreatic NENLM versus rectal NENLM.

In this study, we investigated the efficacy and safety of TAE therapy for 102 times in 32 patients with NENLM at our hospital. We found that TAE significantly improved PFS for those patients, exhibiting better outcomes when used on pancreatic than rectal NENLM.

## 2 MATERIALS AND METHODS

### 2.1 Study design and patients

This cohort study was approved by the Ethics Committee of the First Affiliated Hospital with Nanjing Medical University. The patient and family members signed an informed consent form for TAE. Patients with NENLM who received TAE in our hospital from January 2018 to March 2021 were included. We followed up with the patients by telephone for 1–5 years. They had nonfunctional NENs.

Inclusion criteria were as follows: (1) NENs and NENLM were confirmed by pathological and immunohistochemical examinations; (2) measurable lesions in type III NENLM without indication for surgery discussed by our multidisciplinary team; (3) performance status score of 0–1 with Child–Pugh grade A; (4) NENLM did not invade large blood vessels; (5) contrast‐enhanced computer tomography (CT) or magnetic resonance imaging (MRI) was completed before and after TAE.

Exclusion criteria were as follows: (1) clinical data were not complete, such as missed imaging data or other important therapy; (2) transcatheter arterial chemotherapy embolization (TACE), ablation and other local liver treatments had been performed before TAE except for surgery; (3) contraindication to TAE, such as severe heart, lung, liver or kidney dysfunction.

### 2.2 Characteristics of the patients

Clinical data included age, sex, primary tumour site, grade according to WHO, surgery history of NENLM and other systemic therapies, such as long‐acting octreotide. For evaluation of blood supply, if the density of the NENLM was lower than that of the surrounding normal liver parenchyma in the arterial phase of contrast‐enhanced CT/MRI, it could be considered a hypovascular type.^15^ Hepatic tumour burdens of low (<25%), middle (25%–50%) and high (>50%) levels were classified as cut‐off points according to tumour volume divided by CT as previously reported.^7^, ^8^

### 2.3 Evaluation

**FIGURE 1:**
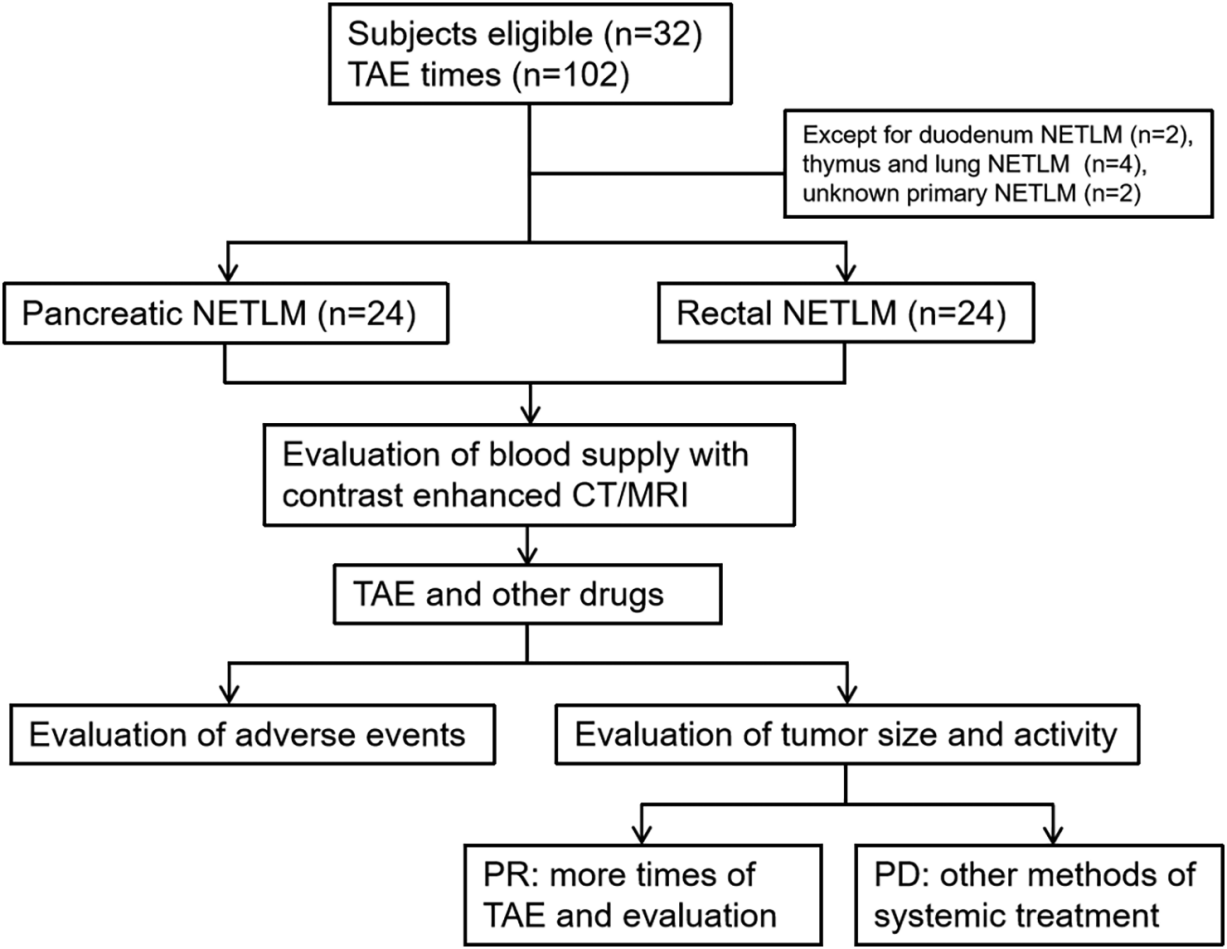
Study flow diagram showed the group allocation.

Tumour shrinkage, objective response rate (ORR), disease control rate (DCR) and progression‐free survival (PFS) were calculated to evaluate the effect of TAE on NENLM and to compare subgroups according to the Response Evaluation Criteria in Solid Tumours (RECIST 1.1). The primary endpoints were ORR, DCR and PFS. Efficacy evaluations are divided into complete response (CR), partial response (PR), stable disease (SD) and disease progression (PD). Objective response rate was defined as the rate of CR plus PR. Disease control rate was defined as the rate of CR, PR and SD. Progression‐free survival was defined as the time from TAE to disease progression. The patients underwent enhanced CT or MRI examinations within 7 days before TAE as baseline data. The same imaging examination was performed within 1–3 months after TAE; subsequently, it was followed up every 3 months (Figure 1).

To explore the key factors influencing the effect of TAE in treating NENLM, we performed univariate and multivariate analyses. Post‐TAE adverse events (AEs) were recorded according to the Common Terminology Criteria for Adverse Events version 5.0 (CTCAE v5.0) of the National Cancer Institute.

### 2.4 Treatment

According to the guidelines, systemic therapy of NENs was continued. A total of 24 patients received long‐acting octreotide 30 mg intramuscular‐injection every 4 weeks, and the remaining patients received oral capecitabine and temozolomide for chemotherapy or everolimus 5–10 mg every day for targeted therapy.

For TAE, we performed digital subtraction angiography (DSA) to assess tumour blood supply and tumour staining. A 3F microcatheter was catheterized into the tumour supply artery. Superselective embospheres of 40–120 μm diameter (Merit Medical Systems) were inserted following the microcatheter according to guidelines. The endpoint of embolization was the stagnation of the secondary branch of the hepatic artery in the area of blood supply of NENLM (the contrast medium was not clear for five cardiac cycles). All patients were treated with short‐acting somatostatin analogs from 1 h before TAE to 48 h after TAE.

### 2.5 Statistical analysis

Statistical analysis was performed using SPSS Statistics version 22. Quantitative data are described as the mean ± standard deviation or median (interquartile range) and were compared using the *t*‐test or the rank‐sum test. Qualitative data were described by numbers and percentages and compared using the χ^2^ or Fisher's exact test. Univariate analysis was performed to identify factors associated with PFS. A Cox proportional hazards regression model was used to identify independent prognostic factors associated with PFS. Survival analysis was carried out by the Kaplan–Meier method. Events were defined by disease progression. All statistical significance was defined as *p* < 0.05.

## 3 RESULTS

### 3.1 Characteristics of the cohort

Thirty‐two patients receiving 102 TAE treatments were included in our study, with an average of 3.19 times per case. Primary sites were the rectum (12 cases, 37.5%), pancreas (12 cases, 37.5%), duodenum (two cases, 6.25%), thymus and lung (four cases, 12.5%) and the unknown (two cases, 6.25%). The average age was 51.59, and 17 (53.1%) were men. There were three (9.4%), 23 (71.9%) and six (18.7%) patients with G1, G2 and G3 tumours, respectively, with a median Ki‐67 (interquartile range, IQR) of 10 (5, 17.5). Three patients (9.4%) received NENLM surgery before TAE. Eight (25%) patients were hypervascular of NENLM, and 24 (75%) cases were hypovascular. Low (<25%), middle (25–50%) and high (>50%) hepatic tumour burdens were found in 13 (40.6%), eight (25%) and 11 (34.4%) patients, respectively.

For subgroup analysis, there were seven patients with hypervascularity in the pancreatic NENLM group and no patients with hypervascularity in the rectal NENLM group (*p* = 0.005). Other characteristics, including age (*p* = 0.06), sex (*p* = 0.22), Ki‐67 (*p* = 0.19), grade (*p* = 0.67), liver surgery history (*p* = 0.59), tumour burden (*p* = 0.83) and long‐acting octreotide therapy (*p* = 0.5), did not show significant differences between the pancreatic NENLM and rectal NENLM groups (Table 1).

**TABLE 1:** Basic characteristics of all patients.

	All NENs	pNENs	rNENs	*p* Value^a^
Age (years)	51.59 ± 10.18	46.33 ± 11.54	43.42 ± 4.56	0.06
Sex (male: female)	17:15	8:4	4:8	0.22
*Primary location*				
Pancreas	12 (37.5%)	NA	NA	NA
Rectum	12 (37.5%)	NA	NA	NA
Duodenum	2 (6.25%)	NA	NA	NA
Thymus and lung	4 (12.5%)	NA	NA	NA
Unknown	2 (6.25%)	NA	NA	NA
Ki‐67 index [IQR]	10 (5, 17.5)	10 (5, 22.5)	9 (5, 12.5)	0.19
*Grade*				
G1	3 (9.4%)	1 (8.3%)	1 (8.3%)	0.67
G2	23 (71.8%)	10 (83.4%)	8 (66.7%)	
G3	6 (18.8%)	1 (8.3%)	3 (25%)	
*Liver surgery*				
Yes	5 (15.6%)	1 (8.3%)	3 (25%)	0.29
No	27 (84.4%)	11 (91.7%)	9 (75%)	
*Blood supply of NENLM*				
Rich	8 (25%)	7 (58.3%)	0	0.005
Poor	24 (75%)	5 (41.7%)	12	
*Tumour burden of liver*				
<25%	13 (40.6%)	4 (33.3%)	5 (41.7%)	0.83
25%–50%	8 (25%)	3 (25%)	2 (16.7%)	
>50%	11 (34.4%)	5 (41.7%)	4 (33.3%)	
*Therapy of long‐acting octreotide*				
Yes	24 (75%)	11 (91.7%)	10 (83.4%)	0.5
No	8 (25%)	1 (8.3%)	2 (16.7%)	

Abbreviations: IQR, interquartile range; NENLM, liver metastasis of NENs; NENs, neuroendocrine neoplasms; pNENs, pancreatic NENs; rNENs, rectal NENs.

^a^ *p* Value compares pancreatic NENLM vs rectal NENLM.

### 3.2 Effect of TAE

Among the 32 cases, no patient achieved CR. There were 12 patients (37.5%) who achieved PR, 12 patients (37.5%) with SD, eight patients (25%) with PD and 16 patients (50%) with tumour shrinkage. The PFS was 12 months (Figure 1A), with an ORR of 37.5% and a DCR of 75%. Seven patients (21.9%) died at the end of this study. In the subgroup analysis, the ORR was 33.3% in the pancreatic NENLM group and 41.7% in the rectal NENLM group (*p* = 0.67), while the DCR was 75% in the pancreatic NENLM group and 66.7% in the rectal NENLM group (*p* = 0.5). In the pancreatic NENLM group, the risks of PD (*p* = 0.09), tumour shrinkage (*p* = 0.07), and death (*p* = 0.19) were better than in the rectal NENLM group. Five patients died in the rectal NENLM group, while two died in the pancreatic NENLM group (Table 2).

**TABLE 2:** Clinical outcomes of TAE in patients with NENLM.

	No. (%)			
	All NENs	pNENs	rNENs	*p* Value^a^
Complete response	0	0	0	NA
Partial response	12 (37.5%)	4 (33.3%)	5 (41.7%)	0.5
Stable disease	12 (37.5%)	5 (41.7%)	3 (25%)	0.33
Disease progression	8 (25%)	1 (8.3%)	4 (33.3%)	0.09
Tumour shrinkage	16 (50%)	6 (50%)	5 (41.7%)	0.07
Objective response rate	12 (37.5%)	4 (33.3%)	5 (41.7%)	0.67
Disease control rate	24 (75%)	9 (75%)	8 (66.7%)	0.5
Progression‐free survival	12 m	NR	7 m	0.01
Death	7 (21.9%)	2 (16.7%)	5 (41.7%)	0.19

^a^ *p* Value compares pancreatic NENLM vs rectal NENLM.

**FIGURE 2:**
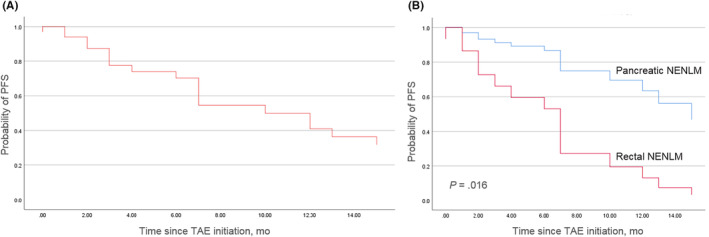
PFS of TAE in NENLM. (A) PFS of all patients. (B) Comparison of PFS between pancreatic NENLM and rectal NENLM. NENLM, neuroendocrine neoplasms with liver metastasis; PSF, progression‐free survival; TAE, transhepatic artery embolization.

Progression‐free survival was not reached in the pancreatic NENLM group, which was significantly better than in the rectal NENLM group (7 months, hazard ratio, 0.22; 95% confidence interval (CI), 0.07–0.76; *p* = 0.016; Figure 2A,B). With univariate and multivariate analyses, we also found that primary pancreatic or rectal sites were the key factor associated with PFS of NENLM (Table 3).

**TABLE 3:** Univariable and multivariable cox regression analyses of factors associated with PFS in patients with pancreatic NENLM vs rectal NENLM.

	Univariable analysis		Multivariable analysis	
	HR (95%CI)	*p* Value	HR (95%CI)	*p* Value
Age (years)	0.4 (0.14–1.15)	0.09	6.8 (0.96–48.76)	0.06
Sex (male: female)	0.37 (0.12–1.11)	0.08	0.53 (0.1–2.78)	0.45
*Primary location*				
Pancreas	1 [Reference]	0.01	1 [Reference]	0.01
Rectum	0.22 (0.07–0.76)		0.22 (0.07–0.76)	
*Grade*				
G1	1 [Reference]	NA	1 [Reference]	NA
G2	0.27 (0.03–2.39)	0.24	0.173 (0.01–2.6)	0.2
G3	0.63 (0.06–6.24)	0.69	0.3 (0.01–11.12)	0.52
*Liver surgery*				
Yes	1 [Reference]	0.2	1 [Reference]	0.6
No	2.41 (0.62–9.36)		1.82 (0.18–17.96)	
*Blood supply of NENLM*				
Rich	1 [Reference]	0.07	1 [Reference]	0.07
Poor	0.25 (0.06–1.11)		0.11 (0.01–1.28)	
*Tumour burden of liver*				
<25%	1 [Reference]	NA	1 [Reference]	NA
25%–50%	0.6 (0.12–3)	0.54	0.61 (0.07–5.5)	0.66
>50%	1.2(0.4–3.6)	0.74	1.88 (0.36–9.83)	0.45
*Therapy of long‐acting octreotide*				
Yes	1 [Reference]	0.42	1 [Reference]	0.74
No	0.59 (0.16–2.13)		0.52 (0.01–26.46)	

**FIGURE 3:**
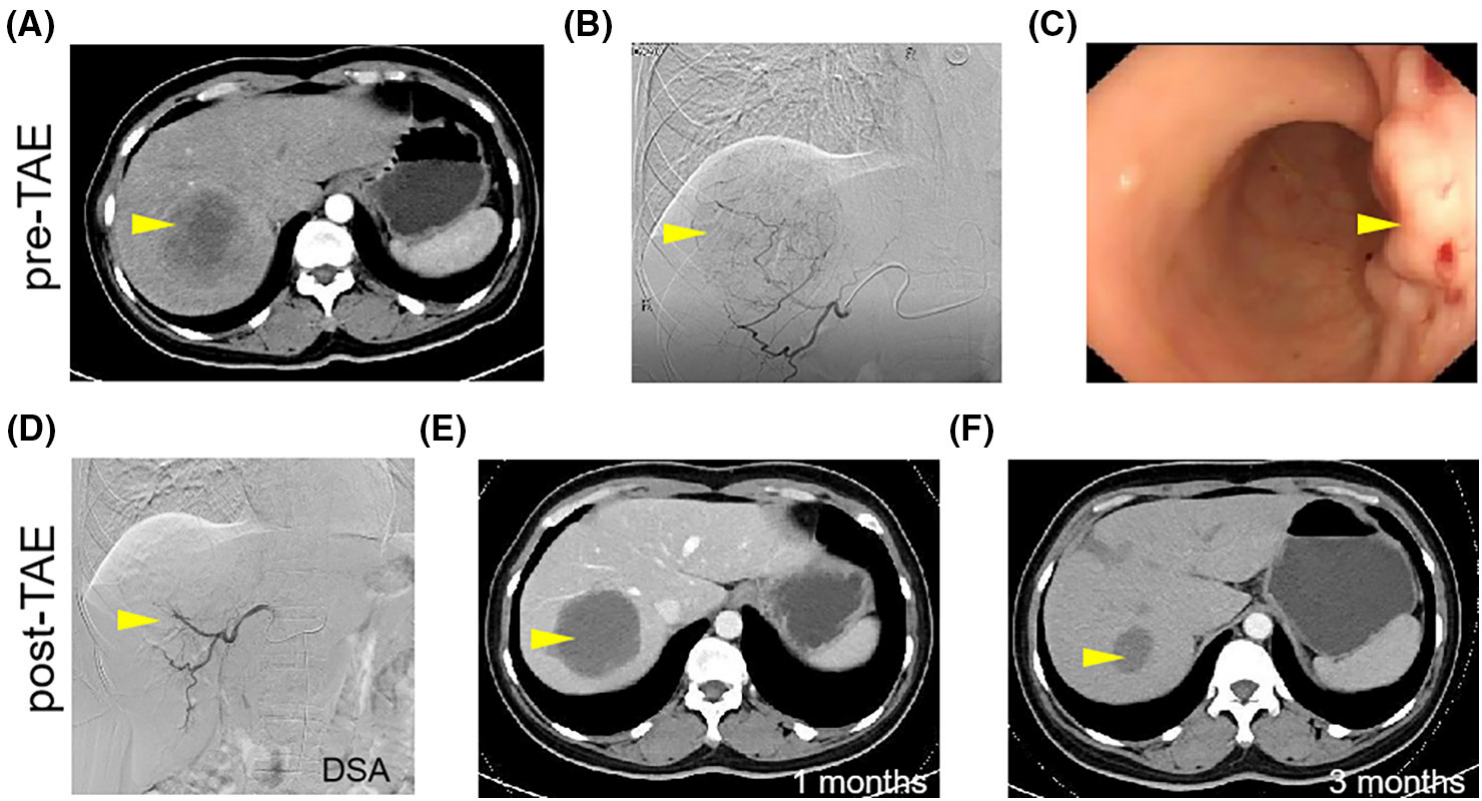
Effect of TAE therapy in a patient with rectal NENLM. (A, B) A patient with rectal NENLM type III with hypovascularism was evaluated by contrast‐enhanced CT and DSA before TAE. (C) Primary rectal NENs were observed under endoscopy. (D, E) The main blood vessels of patients with NENLM were blocked, allowing the tumour to liquefy, necrotic, and shrink. (F) The tumour had shrunk significantly 3 months later by CT examination. Yellow arrows indicated NENLM lesions. CT, computer tomography; DSA, digital subtraction angiography; NEN, neuroendocrine neoplasm; NENLM, neuroendocrine neoplasms with liver metastasis; TAE, transhepatic artery embolization.

We cited two patients with TAE treatment as examples. The first was rectal NENLM type III with hypovascular evaluation by contrast‐enhanced CT and DSA (Figure 3A–C, yellow arrow). After TAE, the main blood vessels of NENLM were blocked, allowing the tumour to liquefy, necrotic and shrink (Figure 3D,E, yellow arrow). The CT results of the follow‐up 3 months later showed that the tumour had shrunk significantly (Figure 3F, yellow arrow).

**FIGURE 4:**
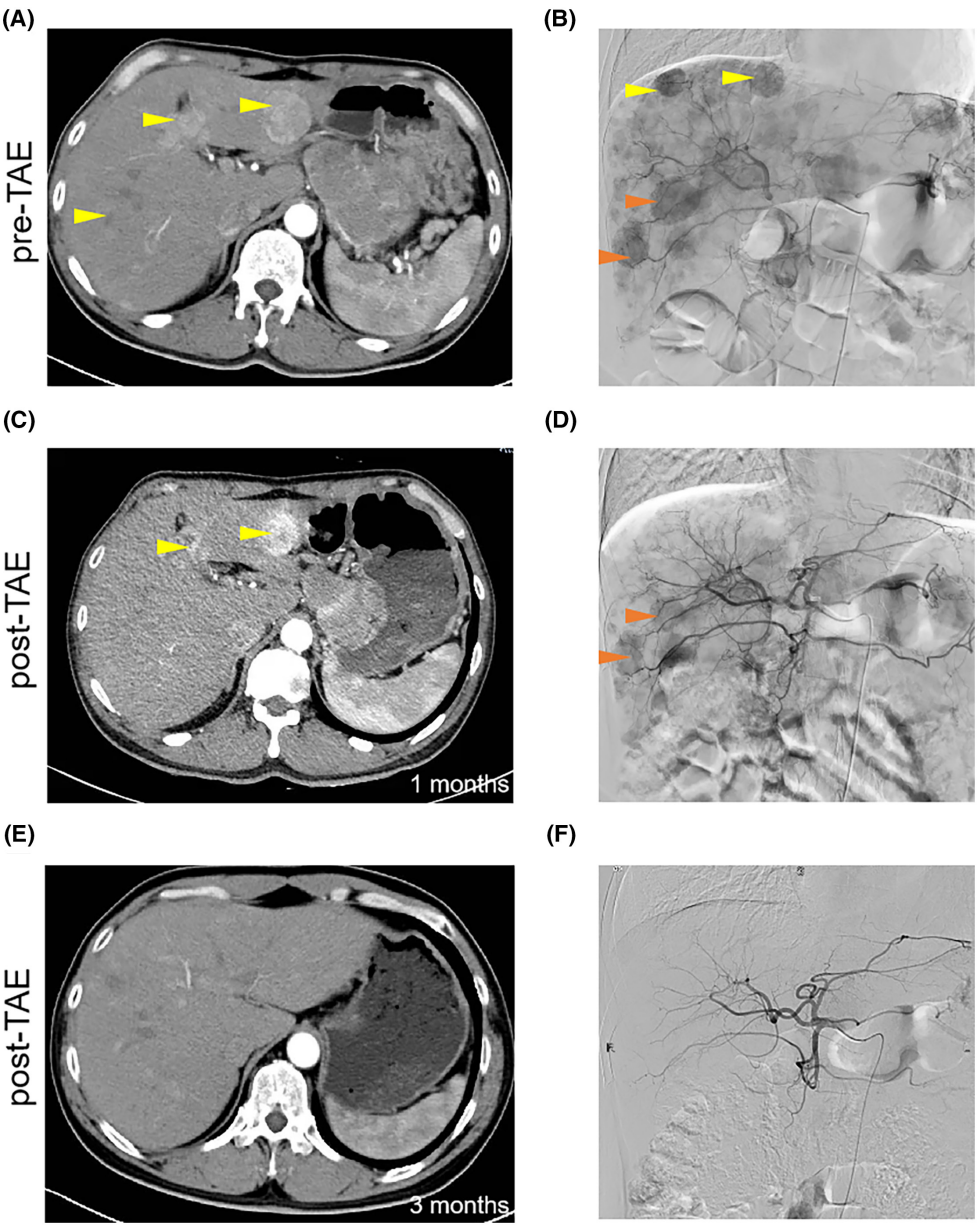
Effect of TAE therapy in a patient with pancreatic NENLM. (A, B) The other case was a patient with type III pancreatic NENLM with hypervascularity evaluated by contrast‐enhanced CT and DSA. (C, D) The main blood vessels of patients with NENLM indicated by the yellow arrows were blocked 1 month later, and the tumour shrank, even including the lesions indicated by the orange arrows. (E, F) All NENLM had disappeared by CT examination 3 months later, and the evaluation of the effect reached CR. The yellow and orange arrows indicated the NENLM lesions. CR, complete response; CT, computer tomography; DSA, digital subtraction angiography; NENLM, neuroendocrine neoplasms with liver metastasis; TAE, transhepatic artery embolization.

The second case was type III pancreatic NENLM with hypervascularity evaluated by contrast‐enhanced CT and DSA (Figure 4A,B, yellow arrows). Due to the numerous lesions, we performed TAE treatment twice to reduce side effects. The lesions indicated by the yellow arrows were treated with the first TAE. After 1 month, the main blood vessels of NENLM, indicated by the yellow arrows, were blocked, and the tumour shrank, even including the lesions indicated by the orange arrows (Figure 4C,D). Transhepatic artery embolization treatment was repeated for the lesions indicated by the orange arrows. Three months later, all NENLMs had disappeared on CT examination, and the evaluation of the effect reached CR (Figure 4E,F).

### 3.3 Adverse effects

The most common AE was abdominal pain (71.9%), followed by liver damage (50%), vomiting (28.1%) and fever (28.1%). Grade 3 or 4 AEs were abdominal pain (6.3%) and elevation of alanine aminotransferase (6.3%), but two patients developed thrombocytopenia, and one patient developed a liver abscess that prolonged the hospitalization period. We need to pay special attention to the higher incidence of grade 3/4 AEs in the pancreatic NENLM group (41.7%) than in the rectal NENLM group (16.7%). All patients with AEs improved and were discharged after symptomatic treatment. There were no significant differences in the incidence of adverse reactions between the pancreatic NENLM group and the rectal NENLM group (Table 4).

**TABLE 4:** Adverse effects during and after TAE.

	No. (%)		
	All NENs	pNENs	rNENs
*Any grade 2–4 toxic effect*			
Abdominal pain	23 (71.9%)	9 (75%)	10 (83.3%)
Vomit	9 (28.1%)	3 (25%)	6 (50%)
Fever	9 (28.1%)	4 (33.3%)	4 (33.3%)
Alanine aminotransferase elevation	16 (50%)	5 (41.7%)	6 (50%)
Thrombocytopenia	2 (6.3%)	2 (16.7%)	0
Hepatic abscess	1 (3.1%)	1 (8.3%)	0
*Grade 3 or 4 toxic effect*			
Abdominal pain	2 (6.3%)	2 (16.7%)	0
Vomit	1 (3.1%)	0	1 (8.3%)
Alanine aminotransferase elevation	2 (6.3%)	0	1 (8.3%)
Thrombocytopenia	2 (6.3%)	2 (16.7%)	0
Hepatic abscess	1 (3.1%)	1 (8.3%)	0

## 4 DISCUSSION

The uniqueness of this cohort study is that the comparative analysis showed that TAE has a better benefit from PFS in relatively hypervascular pancreatic NENLM than hypovascular rectal NENLM. However, TAE treatment of rectal NENLM had a higher risk of progression and death than pancreatic NENLM, which is worth noting in future clinical treatment decisions.

Previous studies on interventional embolization treatment for NENLM have a relatively wide range. Some studies explored the effect of TAE combined with sunitinib in pancreatic NENLM and small intestinal NENLM.^16^ Another study demonstrated the effect of TACE or drug‐eluting beads explained in NENLM.^17^ The differences between TAE and TACE were also compared in a previous study.^18^, ^19^

These studies were mainly based on foreign reports. In their studies, there were more cases of small intestine, which may be directly associated with the relatively high incidence of small intestinal NENs in their countries.^2^, ^20^ In this study, more cases with pancreatic and rectal NENs were included, which have not been reported in the past, especially in the rare study of rectal NENs.

The principle of TAE treatment is to block the blood supply of NENLM to cause tumour ischemia and hypoxia necrosis.^21^, ^22^ Therefore, the blood supply of NENLM should be closely related to the therapeutic effect of TAE. In this study, pancreatic NENLM was rich in blood supply (58.3%), but NENLM originating primarily from other sites, such as the rectum, duodenum, thymus and lung, often showed more hypovascular characteristics, even accounting for 95%, without recommended local effective treatment methods. Is there a therapeutic effect of TAE on hypovascular NENLM, such as rectal NENLM?

In the subgroup analysis of our study, the ORRs were 33.3% and 41.7% in the pancreatic NENLM group and the rectal NENLM group, while the DCRs were 75% and 66.7%, and tumour shrinkage was 50% and 41.7%, respectively. However, PFS was not reached in the pancreatic NENLM group, which was significantly better than in the rectal NENLM group (7 months). It should be noted that PD occurred in one and two patients in the pancreatic and rectal NENLM group, with two and five patients having died, respectively, which indicated a higher risk of progression and death in the rectal NENLM group.

Some reports on the effect of interventional embolization in NENLM exist, but most are based on TACE and transarterial radioembolization (TARE).^23^, ^24^ The largest cohort study of TACE in 122 patients reported a median PFS of 10 months.^25^ Regarding TAE, ORR and PFS have varied widely in previous studies. In our research, the PFS of all patients was 12 months, with an ORR of 37.5%, tumour shrinkage of 50%, and DCR of 75%, indicating that TAE is an effective treatment for NENLM.

The incidence of AEs was similar to that in previous studies.^19^, ^26^ Abdominal pain was the most common (71.9%), followed by liver damage (50%). The main adverse reaction of grade 3 or 4 was abdominal pain (6.3%), but two patients developed thrombocytopenia, and one patient developed a liver abscess (without diabetes and the use of everolimus). The incidence of AEs in the pancreatic NENLM group was higher than in the rectal NENLM group. Although the patients improved and were discharged after active treatment, this would be an important point to be noted, especially more careful observation during TAE treatment in patients with pancreatic NENLM.

Drug treatment remains the main treatment for hypovascular NENLM, and the effect of local treatment has yet to be affirmed. However, NENLM usually has large lesions and tumour load, which seriously inhibits the effect of drug treatment and leads to a poor prognosis for patients. Therefore, we designed this study to observe whether TAE is effective in the local treatment of hypovascular NENLM. In our study, the treatment with TAE of NENLM is safe and effective, even in hypovascular NENLM. Since NENs are relatively rare, the sample size of this study is small. In future, large‐scale clinical studies are needed to explore this issue, and longer follow‐up periods are needed to support the findings of this study.

## AUTHOR CONTRIBUTIONS

**Jian'an Bai:** Conceptualization (supporting); investigation (lead); methodology (supporting); resources (supporting); validation (supporting); writing – original draft (lead). **Jinhua Song:** Data curation (supporting); formal analysis (supporting); funding acquisition (supporting); investigation (lead); methodology (lead); resources (supporting); validation (supporting). **Yang Zhang:** Data curation (supporting); investigation (supporting); project administration (supporting); resources (supporting); software (supporting); writing – original draft (supporting). **Xiaolin Li:** Conceptualization (supporting); resources (supporting); validation (supporting); writing – review and editing (supporting). **Lijun Yan:** Data curation (supporting); investigation (supporting); resources (supporting); software (supporting). **Ping Hu:** Data curation (supporting); formal analysis (supporting); investigation (supporting); methodology (supporting). **Qiyun Tang:** Conceptualization (lead); data curation (lead); funding acquisition (lead); supervision (lead); writing – review and editing (lead).

## CONFLICT OF INTEREST STATEMENT

There is no conflict of interest to declare.

## ETHICS STATEMENT

This cohort study was approved by the Ethics Committee of the First Affiliated Hospital with Nanjing Medical University. The patient and family members signed an informed consent form for TAE. All procedures followed the Declaration of Helsinki for research involving human subjects.

## Data Availability

The data supporting the findings of this study are available upon request from the corresponding author. These data are not publicly available due to privacy or ethics restrictions.
